# Biosignal and Imaging Informatics: 2024 Review and Emerging Trends

**DOI:** 10.1055/s-0046-1816600

**Published:** 2026-11-03

**Authors:** Christian Baumgartner, Leticia Rittner, Thomas M. Deserno

**Affiliations:** 1 Institute of Health Care Engineering with European Testing Center of Medical Devices, Graz Univer-sity of TechnologyAustria; 2 Medical Imaging Computing Lab (MICLab), School of Electrical and Computer Engineering, University of CampinasBrazil; 3 Peter L. Reichertz Institute for Medical Informatics of TU Braunschweig and Hannover Medical SchoolBraunschweigGermany

**Keywords:** Biosignal Informatics, Imaging Informatics, Deep Learning, Spiking Neural Networks, Vision-Language Models, Clinical Integration, Medical AI, Personalized Medicine, IMIA Yearbook

## Abstract

**Objectives:**

This review identifies and showcases key research contributions and emerging trends in Biosignal and Imaging Informatics (BII) published in 2024. The review focus is on innovative studies demonstrating translational potential, computational rigor, and clinical relevance.

**Methods:**

A structured literature search was conducted in PubMed and Scopus, targeting publications up to January 2025. Queries were designed using domain-specific MeSH terms and keywords related to biosignal (sensors and signals) and imaging informatics. Only journals with at least three relevant articles were considered to ensure quality. After removing duplicates, titles and abstracts were assessed independently using a three-point Likert scale. Fourteen high-scoring papers underwent full-text evaluation, and finally, twelve were reviewed by external reviewers and editors. From this process, two standout papers—one from each subfield—were selected as the best contributions.

**Results:**

Out of 399 reviewed articles, twelve were shortlisted for external evaluation. The selected best papers exemplify beyond state-of-the-art innovation: a spiking neural network model for low-power, real-time detection of sleep apnea from single-lead ECG signals, and a universal vision-language model for organ segmentation and tumor detection in abdominal CT imaging. Other notable works addressed challenges in privacy-preserving learning, efficient signal modeling, annotation uncertainty, and real-time clinical deployment, highlighting the field’s evolution toward integrative and practical solutions.

**Conclusion:**

The 2024 BII review reflects a clear transition from algorithmic exploration to clinically viable implementations. Featured studies emphasize not only high-performing machine learning and deep learning techniques, but also address critical issues such as energy efficiency, data privacy, gen-eralizability, and interpretability. Together, they represent the maturing landscape of BII, where com-putational advances increasingly align with the needs of modern, patient-centered healthcare.

## 1. Introduction

Biosignals and Imaging Informatics (BII) remains a dynamic and interdisciplinary area of research, integrating advanced computational methodologies with physiological and medical data analysis. The 2024 review reflects a continued expansion of machine learning (ML) and deep learning (DL) applica-tions across both biosignal processing and medical imaging. The candidate papers selected this year collectively illustrate a shift from theoretical model development toward practical, privacy-preserving, and real-time clinical implementations.

In addition, the BII section of the IMIA Yearbook 2025 presents the commentary paper entitled “Mo-deling Biological Systems: Trends in Biosystem Identification and Biosignal Coupling” by Kostoglou & Müller-Putz [ 1 ]. This work provides a comprehensive overview of current trends and emerging methodologies in biosystem identification and biosignal coupling, emphasizing their roles in under-standing complex physiological dynamics. A key strength of the work lies in its integration of ad-vanced time-varying, nonlinear modeling techniques with modern machine learning and deep learning frameworks. The authors effectively highlight practical applications in real-time monitoring and per-sonalized medicine, showcasing the translational potential of these methods. While recognizing current limitations, the paper underscores the promise of continued computational and technological ad-vancements in addressing clinical and biomedical challenges.

## 2. Paper Selection Process

As in previous years, we restricted our search to English-language articles and excluded review papers to streamline the selection process. Literature searches were conducted in PubMed and Scopus data-bases up to the end of January 2025 (see Appendix B). For biosignal (sensors & signals) and imaging informatics, the searches yielded 106, 36, 416 articles from PubMed, and 7, 26, 139 from Scopus, respectively. To focus on high-impact sources, only journals with at least three publications in the respective subject areas were included [ 2 – 4 ]. After duplicate removal, a total of 399 articles remained (81 and 318 in the respective categories).

The section co-editors independently evaluated titles and abstracts using a three-point Likert scale (1 = not included, 2 = maybe included, 3 = included). Fourteen papers with a cumulative score of seven or higher were selected for full-text review. Using the same criteria, the section co-editors ultimately chose twelve papers for external evaluation, six are from the field of biosignal informatics and six from imaging informatics. Seven external reviewers assessed these papers. Based on their and the co-editors’ rankings, we selected two papers for the 2025 IMIA Yearbook—one in Biosignal Informatics and one in Imaging Informatics ( Table 1 ).

**Table 1:** Selection result of best papers for the 2025 IMIA Yearbook of Medical Informatics for the Biosignal and Imaging Informatics section. A content summary of these best papers can be found in the appendix A of this synopsis.

Section Biosignal and Imaging Informatics (BII)
• Tyagi PK, Agrawal D. Automatic detection of sleep apnea from a single-lead ECG signal based on spiking neural network model. Comput Biol Med. 2024 Sep;179:108877. https://doi.org/10.1016/j.compbiomed.2024.108877 . • Liu J, Zhang Y, Wang K, Yavuz MC, Chen X, Yuan Y, Li H, Yang Y, Yuille A, Tang Y, Zhou Z. Universal and extensible language-vision models for organ segmentation and tumor detection from abdominal computed tomography. Med Image Anal. 2024 Oct;97:103226. https://doi.org/10.1016/j.media.2024.103226 .

## 3. Outlook

### 3.1. Biosignal Informatics

In the biosignal domain, Gao *et al.* [ 5 ] presented a DL-enhanced optical fiber sensor system for con-tinuous monitoring and reconstruction of vital signs, demonstrating the integration of hardware-level sensing with intelligent signal correction. Tyagi and Agrawal [ 6 ] employed a spiking neural network for the detection of sleep apnea using single-lead ECG signals, highlighting the growing interest in neuromorphic computing for low-power, edge-based applications. Weimann and Conrad [ 7 ] explored federated learning in ECG classification, providing a privacy-preserving framework that maintains performance while avoiding centralized data aggregation. Additional studies by Fu *et al.* [ 8 ] and Niu *et al.* [ 9 ] introduced advanced learning frameworks—such as global-local representation fusion and EEG microstate analysis—for emotion recognition and gender classification, respectively, thereby expanding the application of BII to affective and demographic computing. In particular, Niu *et al.* [ 9 ] demonstrated that EEG microstate dynamics exhibit significant gender-specific differences and can serve as reliable neurophysiological biomarkers for gender classification, achieving up to 95.2% accu-racy using machine learning. Proposed by Kim and Choi [ 10 ], a hierarchical model for ECG-based biometric identification was developed, underlining the potential of biosignal data in secure authenti-cation systems.

### 3.2. Imaging Informatics

In imaging informatics, Liu *et al.* [ 11 ] developed a universal vision-language model capable of organ segmentation and tumor detection in abdominal CT scans, aligning with the broader movement toward generalizable multimodal architectures [ 11 ]. Ou *et al.* [ 12 ] introduced RTSeg-net, a lightweight DL model designed for real-time segmentation of fetal head and pubic symphysis in intrapartum ultra-sound, emphasizing computational efficiency and clinical applicability. Proposed by Zwijnen *et al.* [ 13 ], a self-adaptive DL-based segmentation framework for both clinical and preclinical CT imaging was introduced, supporting cross-context deployment. Liao *et al.* [ 14 ] addressed label uncertainty by modeling annotator preference and stochastic errors in medical image segmentation, thereby enhancing annotation reliability. The ATOMMIC toolbox, introduced by Karkalousos *et al.* [ 15 ], facilitate end-to-end consistency in artificial intelligence (AI) pipelines for magnetic resonance imaging (MRI). Last but not least, Devi *et al.* [ 16 ] presented a method for explainable reverse verification of MRI classification results, allowing clinical experts to assess and validate model outputs, and contributing to the growing demand for interpretable AI in medicine.

### 3.3. Future Directions

Collectively, these studies in this review highlight the convergence of ML and DL innovation with clinically oriented requirements, including generalizability, interpretability, efficiency, and privacy. The 2024 body of work reflects a maturing BII landscape, wherein computational models are increas-ingly designed for integration into real-world healthcare settings. As the delineations between signal acquisition, algorithmic modeling, and clinical decision-making continue to dissolve, BII is solidifying its role as a foundational component in the future of intelligent, data-driven medicine.

## 4. Discussion

The 2025 review of Biosignal and Imaging Informatics (BII) underscores the field’s continued evolu-tion toward clinically relevant, real-world applications, driven by advances in machine learning (ML), deep learning (DL), and computational modeling. This year’s selection process highlighted a balanced representation of biosignal and imaging informatics research, with six candidate papers from each domain. A notable trend is the growing emphasis on translational implementations—ranging from edge-computing frameworks for biosignal monitoring to real-time, multimodal imaging models—reflecting a shift from theoretical development toward clinical integration.

Key contributions in biosignal informatics include the application of neuromorphic computing for sleep apnea detection, federated learning for privacy-preserving ECG classification, and EEG microstate analysis for gender classification with high accuracy. These works demonstrate not only algorithmic innovation but also sensitivity to challenges such as energy efficiency, privacy, and biometric security. In parallel, imaging informatics papers showcased generalizable and explainable AI systems, lightweight architectures for real-time ultrasound analysis, and frameworks addressing annotation uncertainty—key requirements for robust clinical deployment.

The invited commentary paper by Kostoglou & Müller-Putz further contextualizes the field’s trajectory by integrating biosystem identification with biosignal coupling, reinforcing the need for sophisticated models that can capture complex physiological interactions. This synthesis of methodological rigor and clinical applicability exemplifies the future direction of BII.

## Biosignals and Imaging Informatics: Appendix

### Queries used for PubMed and Scopus

**Table:** 

PubMed-Sensors
( (“2024/01/01”[DP] : “2024/12/31”[DP]) AND Journal Article [pt] AND English[lang] AND hasabstract[text] NOT Bibliography[pt] NOT Comment[pt] NOT Editorial[pt] NOT Letter[pt] NOT News[pt] NOT Review[pt] NOT Case Reports[pt] NOT Published Erratum[pt] NOT Historical Article[pt] NOT legislation[pt] NOT “clinical trial”[pt] NOT “evaluation studies”[pt] NOT “technical report”[pt] NOT “Scientific Integrity Review”[pt] NOT “Systematic Review”[pt] NOT “Retracted Publication”[pt] ) AND ( ( “sensor”[TI] OR “sensors”[TI] OR “sensing”[TI] ) AND ( “vital sign”[TI] OR “vital signs”[TI] OR “biological signal”[TI] OR “biological signals”[TI] OR “biological parameter”[TI] OR “biological parameters”[TI] OR “physiological parameter”[TI] OR “physiological parameters”[TI] OR “physiological signal”[TI] OR “physiological signals”[TI] OR “blood pressure”[TI] OR “temperature”[TI] OR “heart rate”[TI] OR “heartbeat”[TI] OR “heartbeats”[TI] OR “pulse rate”[TI] OR “respiration rate”[TI] OR “respiratory rate”[TI] OR “breathing rate”[TI] OR “ECG”[TI] OR “elec-trocardiography”[TI] OR “electrocardiogram”[TI] OR “menstrual cycle”[TI] OR “oxygen”[TI] OR “oximetry”[TI] OR “glucose”[TI] OR “end-tidal”[TI] OR “emg”[TI] OR “electromyography”[TI] OR “electromyogram”[TI] OR “ppg”[TI] OR “photoplethysmography”[TI] OR “photoplethysmogram”[TI] OR “pcg”[TI] OR “phonocardiography”[TI] OR “phonocardiogram”[TI] OR “bcg”[TI] OR “ballisto-cardiography”[TI] OR “ballistocardiogram”[TI] OR “scg”[TI] OR “seismocardiography”[TI] OR “seismocardiogram”[TI] OR “eog”[TI] OR “electrooculography”[TI] OR “electrooculogram”[TI] OR “eda”[TI] OR “electrodermal activity”[TI] OR “GSR”[TI] OR “Galvanic skin response” [TI] OR “eeg”[TI] OR “electroencephalogram”[TI] OR “bci”[TI] OR “brain computer interface”[TI] ) NOT ( “review”[TI] OR “survey”[TI] OR “conference”[ta] ) ) AND (“medic*”[TIAB] OR “biomed*”[TIAB] OR “biologic*[TIAB]”)

**Table:** 

Scopus-Sensors
TITLE((“sensor” OR “sensors” OR “sensing”) AND (“vital sign” OR “vital signs” OR “biological signal” OR “biological signals” OR “biological parameter” OR “biological parameters” OR “physiological parameter” OR “physiological parameters” OR “physiological signal” OR “physiological signals” OR “blood pressure” OR “temperature” OR “heart rate” OR “heartbeat” OR “heartbeats” OR “pulse rate” OR “respiration rate” OR “respiratory rate” OR “breathing rate” OR “ECG” OR “elec-trocardiography” OR “electrocardiogram” OR “menstrual cycle” OR “oxygen” OR “oximetry” OR “glucose” OR “end-tidal” OR “emg” OR “electromyography” OR “electromyogram” OR “ppg” OR “photoplethysmography” OR “photoplethysmogram” OR “pcg” OR “phonocardiography” OR “phonocardiogram” OR “bcg” OR “ballistocardiography” OR “ballistocardiogram” OR “scg” OR “seismocardiography” OR “seismocardiogram” OR “eog” OR “electrooculography” OR “electrooculogram” OR “eda” OR “electrodermal activity” OR “GSR” OR “Galvanic skin response” OR “eeg” OR “electroencephalogram” OR “bci” OR “brain computer interface”) AND NOT ( “review” OR “survey” )) AND TITLE-ABS (“application”) AND PUBDATETXT (“January 2024” OR “February 2024” OR “March 2024” OR “April 2024” OR “May 2024” OR “June 2024” OR “July 2024” OR “August 2024” OR “September 2024” OR “October 2024” OR “November 2024” OR “December 2024”) AND LANGUAGE(english) AND SUBJAREA(MEDI) AND SRCTYPE(j) AND DOCTYPE(ar) AND NOT DOCTYPE(re) AND ( EXCLUDE ( SUBJAREA , “ENGI” ) AND ( EXCLUDE ( EXACTKEYWORD , “Controlled Study” ) OR EXCLUDE ( EXACTKEYWORD , “Comparative Study” ) OR EXCLUDE ( EXACTKEYWORD , “Retrospective Study” ) OR LIMIT-TO ( EXACTKEYWORD , “Article” ) )

**Table:** 

PubMed -Signals
((“2024/01/01”[DP] : “2024/12/31”[DP]) AND Journal Article [pt] AND English[lang] AND hasabs-tract[text] NOT Bibliography[pt] NOT Comment[pt] NOT Editorial[pt] NOT Letter[pt] NOT News[pt] NOT Review[pt] NOT Case Reports[pt] NOT Published Erratum[pt] NOT Historical Article[pt] NOT legislation[pt] NOT “clinical trial”[pt] NOT “evaluation studies”[pt] NOT “technical report”[pt] NOT “Scientific Integrity Review”[pt] NOT “Systematic Review”[pt] NOT “Retracted Publication”[pt] ) AND ( ( “biosignal”[TI] OR “biomedical signal”[TI] OR “physiological signal”[TI] OR “ecg”[TI] OR “electrocardiography”[TI] OR “electrocardiogram”[TI] OR “emg”[TI] OR “electromyography”[TI] OR “electromyogram”[TI] OR “ppg”[TI] OR “photoplethysmography”[TI] OR “photoplethysmogram”[TI] OR “pcg”[TI] OR “phonocardiography”[TI] OR “phonocardiogram”[TI] OR “bcg”[TI] OR “ballistocardiography”[TI] OR “ballistocardiogram”[TI] OR “scg”[TI] OR “seismocardiography”[TI] OR “seismocardiogram”[TI] OR “eog”[TI] OR “electrooculography”[TI] OR “electrooculogram”[TI] OR “eda”[TI] OR “electrodermal activity”[TI] OR “Respiration”[TI] OR “Blood Pressure”[TI] OR “eeg”[TI] OR “electroencephalogram”[TI] OR “bci”[TI] OR “brain computer interface”[TI] ) AND ( “processing”[TI] OR “analytics”[TI] OR “analysis”[TI] OR “analyse”[TI] OR “analyze”[TI] OR “analysing”[TI] OR “analyzing”[TI] OR “enhancement”[TI] OR “enhancements”[TI] OR “segmentation”[TI] OR “feature extraction”[TI] OR “feature selection”[TI] OR “classification”[TI] OR “clustering”[TI] OR “measurement”[TI] OR “quantification”[TI] OR “registration”[TI] OR “reco-gnition”[TI] OR “reconstruction”[TI] OR “interpretation”[TI] OR “retrieval”[TI] OR “augmenta-tion”[TI] OR “data mining”[TI] OR “computer-assisted”[TI] OR “computer-aided”[TI] OR “artificial intelligence”[TI] OR “machine learning”[TI] OR “deep learning”[TI] OR “neural network”[TI] OR “computer vision”[TI] OR “autoencoder”[TI] OR “auto-encoder”[TI] OR “Boltzmann”[TI] OR “U-net”[TI] OR “support vector machine”[TI] OR “SVM”[TI] OR “random forest”[TI] ) NOT ( “re-view”[TI] OR “survey”[TI] OR “conference”[ta] ) ) AND ( “medical informatics”[MH] )

**Table:** 

Scopus-Signals
TITLE((“signal” OR “biosignal” OR “biomedical signal” OR “physiological signal” OR “ecg” OR “electrocardiography” OR “electrocardiogram” OR “emg” OR “electromyography” OR “electromyo-gram” OR “ppg” OR “photoplethysmography” OR “photoplethysmogram” OR “pcg” OR “phonocardiography” OR “phonocardiogram” OR “bcg” OR “ballistocardiography” OR “ballistocardiogram” OR “scg” OR “seismocardiography” OR “seismocardiogram” OR “eog” OR “electrooculography” OR “electrooculogram” OR “eda” OR “electrodermal activity” OR “Respiration” OR “Blood Pressure” OR “eeg” OR “electroencephalogram” OR “bci” OR “brain computer interface” ) AND (“processing” OR “analytics” OR “analysis” OR “analyse” OR “analyze” OR “analysing” OR “analyzing” OR “enhancement” OR “enhancements” OR “segmentation” OR “feature extraction” OR “feature selection” OR “classification” OR “clustering” OR “measurement” OR “quantification” OR “registration” OR “recognition” OR “reconstruction” OR “interpretation” OR “retrieval” OR “augmentation” OR “data mining” OR “computer-assisted” OR “computer-aided” OR “artificial intelligence” OR “machine learning” OR “deep learning” OR “neural network” OR “com-puter vision” OR “autoencoder” OR “auto-encoder” OR “Boltzmann” OR “U-net” OR “support vector machine” OR “SVM” OR “random forest”) AND NOT (“review” OR “survey” )) AND PUBDATETXT (“January 2024” OR “February 2024” OR “March 2024” OR “April 2024” OR “May 2024” OR “June 2024” OR “July 2024” OR “August 2024” OR “September 2024” OR “October 2024” OR “November 2024” OR “December 2024”) AND LANGUAGE(english) AND SUBJAREA(MEDI) AND SRCTYPE(j) AND DOCTYPE(ar) AND NOT DOCTYPE(re) AND ( LIMIT-TO ( SUBJAREA , “COMP” ) OR EXCLUDE ( SUBJAREA , “ENGI” ) ) AND ( EXCLUDE ( EXACTKEYWORD , “Controlled Study” ) OR EXCLUDE ( EXACTKEYWORD , “Comparative Study” ) OR EXCLUDE ( EXACTKEYWORD , “Retrospective Study” ) OR LIMIT-TO ( EXACTKEYWORD , “Article” ) )

**Table:** 

PubMed - Imaging
(“2024/01/01”[DP] : “2024/12/31”[DP]) AND Journal Article[pt] AND English[lang] AND hasabs-tract[text] NOT Bibliography[pt] NOT Comment[pt] NOT Editorial[pt] NOT Letter[pt] NOT News[pt] NOT Review[pt] NOT Case Reports[pt] NOT Published Erratum[pt] NOT Historical Article[pt] NOT legislation[pt] NOT “clinical trial”[pt] NOT “evaluation studies”[pt] NOT “technical report”[pt] NOT “Scientific Integrity Review”[pt] NOT “Comparative Study”[PT] NOT “Systematic Review”[pt] NOT “Retracted Publication”[pt] AND (“image”[TI] OR “imaging”[TI] OR “X-ray”[TI] OR “X ray”[TI] OR “MRI”[TI] OR “ultrasound”[TI] OR “endoscopy”[TI] OR “arthroscopy”[TI] OR “tomography”[TI] OR “microscopy”[TI]) AND (“processing”[TI] OR “analytics”[TI] OR “analysis”[TI] OR “segmentation”[TI] OR “classification”[TI] OR “feature extraction”[TI] OR “artificial intelligence”[TI] OR “machine learning”[TI] OR “neural network”[TI] OR “computer vision”[TI]) AND “medical informatics”[MH] NOT (“review”[TI] OR “survey”[TI] OR “conference”[ta])

**Table:** 

Scopus - Imaging
TITLE((“image” OR “imaging” OR “X-ray” OR “X ray” OR “MRI” OR “ultrasound” OR “endoscopy” OR “arthroscopy” OR “tomography” OR “microscopy”) AND (“processing” OR “analy*” OR “seg-mentation” OR “classification” OR “feature extraction” OR “artificial intelligence” OR “machine lear-ning” OR “neural network” OR “computer vision”) AND NOT (“review” OR “survey”)) AND PUB-DATETXT (“January 2024” OR “February 2024” OR “March 2024” OR “April 2024” OR “May 2024” OR “June 2024” OR “July 2024” OR “August 2024” OR “September 2024” OR “October 2024” OR “November 2024” OR “December 2024”) AND LANGUAGE(english) AND SUBJAREA(MEDI) AND SRCTYPE(j) AND DOCTYPE(ar) AND NOT DOCTYPE(re) AND ( LIMIT-TO ( SUBJAREA , “COMP” ) OR EXCLUDE ( SUBJAREA , “ENGI” ) ) AND ( EXCLUDE ( EXACTKEYWORD , “Controlled Study” ) OR EXCLUDE ( EXACTKEYWORD , “Comparative Study” ) OR EXCLUDE ( EXACTKEYWORD , “Retrospective Study” ) OR LIMIT-TO ( EXACTKEYWORD , “Article” ) )
